# The Monofunctional Catalase KatE of *Xanthomonas axonopodis* pv. *citri* Is Required for Full Virulence in Citrus Plants

**DOI:** 10.1371/journal.pone.0010803

**Published:** 2010-05-24

**Authors:** María Laura Tondo, Silvana Petrocelli, Jorgelina Ottado, Elena G. Orellano

**Affiliations:** Molecular Biology Division, Facultad de Ciencias Bioquímicas y Farmacéuticas, Instituto de Biología Molecular y Celular de Rosario, Consejo Nacional de Investigaciones Científicas y Técnicas, Universidad Nacional de Rosario, Rosario, Argentina; University of Wisconsin-Milwaukee, United States of America

## Abstract

**Background:**

*Xanthomonas axonopodis* pv. *citri* (Xac) is an obligate aerobic phytopathogen constantly exposed to hydrogen peroxide produced by normal aerobic respiration and by the plant defense response during plant-pathogen interactions. Four putative catalase genes have been identified *in silico* in the Xac genome, designated as *katE*, *catB*, *srpA* (monofunctional catalases) and *katG* (bifunctional catalase).

**Methodology/Principal Findings:**

Xac catalase activity was analyzed using native gel electrophoresis and semi-quantitative RT-PCR. We demonstrated that the catalase activity pattern was regulated in different growth stages displaying the highest levels during the stationary phase. KatE was the most active catalase in this phase of growth. At this stage cells were more resistant to hydrogen peroxide as was determined by the analysis of CFU after the exposition to different H_2_O_2_ concentrations. In addition, Xac exhibited an adaptive response to hydrogen peroxide, displaying higher levels of catalase activity and H_2_O_2_ resistance after treatment with sub-lethal concentrations of the oxidant. In the plant-like medium XVM2 the expression of KatE was strongly induced and in this medium Xac was more resistant to H_2_O_2_. A Xac*katE* mutant strain was constructed by insertional mutagenesis. We observed that catalase induction in stationary phase was lost meanwhile the adaptive response to peroxide was maintained in this mutant. Finally, the Xac*katE* strain was assayed *in planta* during host plant interaction rendering a less aggressive phenotype with a minor canker formation.

**Conclusions:**

Our results confirmed that in contrast to other Xanthomonas species, Xac catalase-specific activity is induced during the stationary phase of growth in parallel with the bacterial resistance to peroxide challenge. Moreover, Xac catalases expression pattern is modified in response to any stimuli associated with the plant or the microenvironment it provides. The catalase KatE has been shown to have an important function for the colonization and survival of the bacterium in the citrus plant during the pathogenic process. Our work provides the first genetic evidence to support a monofunctional catalase as a virulence factor in Xac.

## Introduction

Aerobic organisms are usually exposed to a variety of reactive oxygen species (ROS) such as the superoxide radical (O_2_^−^), hydrogen peroxide (H_2_O_2_), and the hydroxyl radical (^.^OH), which are produced by the stepwise one-electron reduction of molecular oxygen [1], [2]. Univalent reduction of O_2_ leads to the production of superoxide, which may be rapidly converted to hydrogen peroxide through spontaneous dismutation or via disproportionation by the action of superoxide dismutases (SODs) [1], [3]. The highly reactive hydroxyl radical is generated when hydrogen peroxide reacts with Fe^2+^ in the Fenton reaction, thereby linking cellular iron status to oxidative stress. Thus, exposure to ROS is an unavoidable consequence of aerobic metabolism.

However, ROS are also important components of the host immune response, and many pathogens need to prevent and overcome oxidative stress in order to establish and maintain infections [4]. The use of ROS as antimicrobial agents by the immune system is based on the high reactivity of this species with various cellular components, leading to lesions in DNA, damage to iron-sulphur clusters of key enzymes, oxidation of protein thiols and peroxidation of lipids in the invading bacteria [5], [6].

To cope with the harmful effects of ROS, most aerobic organisms have evolved an arsenal of enzymes involved in either direct detoxification of ROS or repair processes of oxidatively damaged cellular components [2]. Among antioxidant enzymes, catalases (E.E. 1.11.1.6; H_2_O_2_:H_2_O_2_ oxidoreductase) are central components of the detoxification pathways that prevent formation of the highly reactive hydroxyl radical by catalyzing the dismutation of H_2_O_2_ to water and oxygen. Based on their enzymological properties, bacterial catalases have been classified into three types: (*i*) monofunctional heme-containing catalases, further subdivided into the small- and large-subunit categories; (*ii*) bifunctional heme-containing catalase-peroxidases, closely related by sequence and structure to plant peroxidases; and (*iii*) nonheme or Mn-containing catalases [7], [8].

Bacterial catalase levels are largely determined by two factors: the content of H_2_O_2_ in the medium and the entry of cells into the stationary phase of growth [7]. Most bacterial species possess multiple catalase isozymes encoded by different genes; which are regulated differently in terms of growth phase and response to oxidative stress, suggesting that they may have different physiological functions [7], [9].

*Xanthomonas axonopodis* pv. *citri* (Xac) is a Gram negative obligate aerobic bacterium. The organism is also the phytopathogen responsible for citrus canker, a severe disease that affects most commercial citrus cultivars [10], [11].

One of the earliest responses to pathogen recognition in plant defense is the so-called oxidative burst, which consists of the rapid generation of ROS, primarily H_2_O_2_, at the site of attempted invasion [12]. It has been reported that in plant-pathogen incompatible interactions, accumulation of H_2_O_2_ occurs in a biphasic manner, being the second phase of the oxidative response responsible for the establishment of desease resistance. During compatible interactions, in which the pathogen is capable of colonizing a susceptible plant and causing disease, only the first peak of lower magnitude is observed, which appears to be a non-specific plant response to a variety of stress stimuli [13].

The capacity of phytopathogenic bacteria to multiply in host plant tissues may be due, in part, to the ability of these organisms to detoxify H_2_O_2_. In contrast to other active oxygen species, H_2_O_2_ can penetrate through membranes to affect a variety of cellular processes. In this way, catalase is likely very important for Xac in detoxifying H_2_O_2_ generated (*i*) endogenously through normal aerobic respiration, and (*ii*) by the oxidative burst of plant cells during plant-pathogen interactions.

Four genes encoding putative catalase enzymes have been identified in the Xac genome (http://cancer.lbi.ic.unicamp.br/xanthomonas/) [14], [15]. Comparative sequence analysis of their deduced amino acid sequences indicate that *srpA* (XAC3990), *katE* (XAC1211) and *catB* (XAC4029 and XAC4030) genes encode putative monofunctional catalases while *katG* (XAC1301) encodes a bifunctional catalase-peroxidase. In the present study we investigated the Xac response to hydrogen peroxide and studied the expression patterns of the catalase genes during the bacterial growth cycle and in a plant-like medium. A mutant strain lacking a functional *katE* gene was constructed and used to demonstrate that KatE is the major catalase induced in Xac during the stationary phase of growth. Furthermore, the virulence of the mutant strain was assessed during plant-pathogen interactions with host plants revealing the importance of this catalase in the plant colonization process.

## Materials and Methods

### Bacterial strains, plasmids and growth conditions

Bacterial strains and plasmids used in this study are described in Table 1. Xac strains were routinely grown aerobically in Silva Buddenhagen (SB) medium (5 g l^−1^ sucrose, 5 g l^−1^ yeast extract, 5 g l^−1^ peptone, and 1 g l^−1^ glutamic acid, pH 7.0) at 28°C with shaking at 200 rpm, or on 1.5% Bacto agar-SB plates. For the *in vitro* studies of pathogen responses to plant-like media, cells were grown in nutrient broth (NB, 3 g l^−1^ beef extract and 5 g l^−1^ beef peptone) and in the *hrp*-inducing minimal medium XVM2 (20 mM NaCl, 10 mM (NH_4_)_2_SO_4_, 1 mM CaCl_2_, 10 µM FeSO_4_, 5 mM MgSO_4_, 0.16 mM KH_2_PO_4_, 0.32 mM K_2_HPO_4_, 10 mM fructose, 10 mM sucrose and 0.03% (w/v) casein acid hydrolysate (casaminoacid), pH 6.7). *Escherichia coli* strains were grown at 37°C in Luria-Bertani (LB) medium [16]. Antibiotics were added to the media at the following final concentrations: ampicillin (Ap) 100 µg ml^−1^ for *E. coli* and 25 µg ml^−1^ for Xac, kanamycin (Km) 40 µg ml^−1^ and gentamycin (Gm) 40 µg ml^−1^ for *E. coli* and 20 µg ml^−1^ for Xac. Xac strain Xcc99-1330 was kindly provided by Blanca I. Canteros (INTA Bella Vista, Argentina).

**Table 1:** Bacterial strains, plasmids and primers used in this work.

Strain/plasmid	Relevant genotype and description	Source/reference
**Strains**		
*Xanthomonas axonopodis* pv. *citri*		
Xcc99-1330	Wild type, Ap^r^	B. I. Canteros
Xac*katE*	*katE* mutant of Xcc99-1330, Km^r^, Ap^r^	This work
cXac*katE*	Xac*katE* complemented, carries pBBR1/katE, Km^r^, Gm^r^, Ap^r^	This work
*Escherichia coli*		
JM109	*HsdR17 endA1 Recal thi gyrA96 relA1 recA1 supE44 λ^−^Δ(lac-proAB),* [F′, traD36, proA^+^B^+^, *lacI^q^ZΔM15*]	[16]
S17-1	*thi, pro, hsdR, recA* with RP4-2[Tc::Mu-Km::Tn7], Sm^r^	[21]
**Plasmids**		
pGEM-T Easy	PCR cloning and sequencing vector, Ap^r^	Promega
pGEM/*katE*	pGEM-T Easy containing 440-bp fragment of *katE*	This work
pK18mobGII	pUC18 derivative, *lacZa, gusA, mob* site, Km^r^	[24]
pKmob/*katE*	pK18mobGII containing 440-bp fragment of *katE*	This work
pBBR1MCS-5	Broad host-range vector, Gm^r^	[25]
pBBR1/*katE*	pBBR1MCS-5 containing *katE* gene	This work

Primer name	Sequence^a^	Amplified fragment
srpA-F	5′ attggatccGGGCCAATACGCAGTACAAC 3′	432 bp of the XAC3990 gene
srpA-R	5′ aattaagcttGATTGAACGATTGCGAATACAC 3′	
katE-F1	5′ attggatccTCGGTATTCATAGCTTCCGTTT 3′	440 bp of the XAC1211 gene
katE-R1	5′ aattaagcttCGTGTCCAGATACGAGAACAAC 3′	
katE-F2	5′ TGATTCATGCGGTGAAGATGG 3′	887 bp of the XAC1211 gene
katE-R2	5′ GGTCTGGCTACGGAAGAACAG 3′	
ckatE-F	5′ aattaagcttATGACATGGAGGAGCTCTGG 3′	2729 bp including XAC 1211
ckatE-R	5′ attggatccCTTCCCTAGGGCGGCTAC 3′	
katG-F	5′ CTGGTGAAGGACGACGACAG 3′	405 bp of the XAC1301 gene
katG-R	5′ aattaagcttCAGATGGTCGAAGTAATCGTTG 3′	
catB-F	5′ attggatccTTGAACAAGAACGTCGACAACT 3′	492 bp of the XAC4029 gene
catB-R	5′ aattaagcttCTTGTACAGGAACGACAGCATC 3′	
R16S-F	5′ TGGTAGTCCACGCCCTAAACG 3′	217 bp of the XAC4291 gene
R16S-R	C5′ TGGAAAGTTCCGTGGATGTC 3′	

Ap, ampicillin; Km, kanamycin; Gm, gentamycin; Sm, streptomycin.

**a.** Capital letters correspond to nucleotides of the Xac genome sequence and small letters to nucleotides added to facilitate cloning.

### Preparation of soluble cell extracts

Cell extracts were prepared from 10 ml cultures harvested by centrifugation at 10000 *g* for 10 min at 4°C. Bacteria were washed and resuspended in 500 µl of ice-cold 50 mM potassium phosphate buffer (pH 7.0) containing 1 mM PMSF, and then disrupted by intermittent sonication. Suspensions were clarified by centrifugation at 12000 *g* for 20 min at 4°C. Protein concentrations in soluble cell extracts were determined by the method of Sedmack and Grossberg [17] with bovine serum albumin as a standard.

### Enzyme activity assay and staining

Catalase activity in cell extracts was monitored through the decomposition of hydrogen peroxide by following the decrease in absorbance at 240 nm [18]. The assays were performed at 25°C in 50 mM potassium phosphate buffer (pH 7.0), containing 10 mM H_2_O_2_. An extinction coefficient of 43.6 M^−1^ cm^−1^ at 240 nm was used to calculate the specific activity. One unit of catalase activity was defined as the amount of activity required to decompose 1 µmol of H_2_O_2_ per minute under the assay conditions.

For catalase activity staining, aliquots of cell extracts containing 25–50 µg of soluble protein were electrophoresed on 8% non-denaturing polyacrylamide gels and stained for catalase activity as described by Scandalios [19]. To eliminate the likelihood of multiple, potentially artifactual catalase bands, which can be detected at higher amperage (20 to 30 mA), non-denaturing gels were electrophoresed at 10 mA to resolve these bands.

### Recombinant DNA and microbiological techniques

All DNA manipulations including plasmid purification, restriction enzyme digestion, DNA ligation and agarose gel electrophoresis were performed with standard techniques [16]. Total bacterial genomic DNA from Xac was isolated using the cetyltrimethylammonium bromide procedure [20]. Plasmids for bacterial conjugations were transferred to Xac by biparental mating from the broad host-range-mobilizing *E. coli* strain S17-1 [21]. Bacterial mixtures were spotted onto Hybond-C membranes, placed on SB-agar and incubated for 48 h at 28°C. Membranes were then washed and bacteria transferred to selective medium as previously described [22].

### Survival in the presence of hydrogen peroxide

Survival experiments were performed by subculturing Xac overnight cultures into fresh SB medium at 2% inoculum. After 4 or 24 h of growth (early exponential and stationary phase, respectively) aliquots of the cultures were diluted and plated on SB-agar plates. Hydrogen peroxide was then added to the cultures at final concentrations of 0.25 to 30 mM. After 15 min of exposure to the oxidant, samples were removed, washed once with fresh medium, serially diluted and plated on SB-agar plates.

To assess the H_2_O_2_ resistance of Xac in a plant-like medium, Xac overnight cultures were subcultured into fresh NB or XVM2 media [23] at 2% inoculum and grown for 7 or 16 h to early exponential and stationary phase respectively. Survival experiments were then performed as previously described using final concentrations of 1 and 30 mM H_2_O_2_.

For the induction experiments, Xac cultures were grown to early exponential phase and incubated with sub-lethal concentrations of hydrogen peroxide (10, 30 and 100 µM) for an additional hour before being used in the killing experiments. After the induction treatment, aliquots of the cultures were washed, diluted and plated on SB-agar plates. Cultures were then treated with a range of lethal concentrations of H_2_O_2_ (0.25 to 5 mM) for 15 min, after which samples were removed, washed once with fresh medium, serially diluted and plated on SB-agar plates.

In all cases, growth of liquid cultures was monitored spectrophotometrically by optical density at 600 nm (OD_600_). Colonies were counted after 48 h incubation at 28°C. The percentage of survival was defined as the number of colony forming units (CFU) after treatment divided by the number of CFU prior to treatment ×100.

### RNA extraction and semi-quantitative reverse transcription PCR (RT-PCR)

Total RNA of Xac cells was isolated using TRIzol® reagent (Invitrogen), according to the manufacturer's instructions. After extraction, the RNA was treated with RNase-free DNase (Promega) and its integrity was checked by agarose gel electrophoresis. Semi-quantitative analyses of transcript levels of *katE*, *srpA*, *catB* and *katG* were carried out using a two-step RT-PCR approach employing the primers listed in Table 1. For cDNA synthesis, total RNA (1 µg) was added to a 20 µl reverse transcription reaction medium containing 4 µl 5× M-MLV buffer (Promega), 0.5 mM dNTP mixture, 0.5 µg gene-specific primer, 200 U M-MLV reverse transcriptase (Promega) and incubated for 60 min at 42°C. Reverse transcription was terminated by incubating for 5 min at 94°C. Control reactions, where RT was omitted, were done in parallel for all the samples to rule out the possibility of amplification from contaminating DNA. PCR reactions were carried out with 2 µl cDNA template under the following conditions: 25 cycles of denaturation at 94°C for 1 min, annealing at 65°C for 1 min, and extension at 72°C for 1 min; with a final extension step at 72°C for 5 min. The number of cycles to be used, avoiding reaching the plateau of the PCRs, was previously determined by taking samples at different number of cycles during the PCR amplification step and analyzing the products obtained by agarose gel electrophoresis. As a constitutive control, a 217-bp fragment of 16S rRNA was amplified using the same PCR conditions but with only 1% of the cDNA synthesis reaction as template due to the high abundance of 16S rRNA in total RNA extracts. RT-PCR products were resolved on 1.5% (w/v) agarose gels, and densitometrically quantified using Gel-Pro Analyzer Software 3.1 (Media Cybernetics).

### Construction of the Xac*katE* mutant strain

The Xac*katE* mutant was constructed by insertional inactivation of the *katE* gene on the chromosome by a single homologous recombination. Primers katE-F1 and katE-R1 (Table 1), were used to amplify a 440-bp internal fragment of the *katE* coding region using Xac genomic DNA as template. The PCR product was cloned into pGEM-T Easy vector (Promega), and the nucleotide sequence of the insert was confirmed by automated DNA sequencing. Subsequently, a *Hind*III-*Bam*HI fragment of the PCR product was subcloned into pK18mobGII [24], rendering pKmob/*katE* (Table 1). The recombinant plasmid pKmob/*katE* was transferred from *E. coli* strain S17-1 [21] to the Xac wild-type strain by conjugation. Recombination of the cloned *katE* fragment in the suicide plasmid with the homologous counterpart on the Xac chromosome resulted in the disruption of the *katE* gene. The *katE* mutant was selected on SB-agar plates containing 40 µg ml^−1^ Km. Inactivation of *katE* was confirmed by PCR using specific primers katE-F2 and katE-R2, located upstream and downstream of the gene fragment used for the homologous recombination (Table 1).

For mutant complementation, a 2729-bp DNA fragment containing the *katE* coding region and extending from 603 bp upstream of the 5′ end to 14 bp downstream of the 3′ end of the ORF was amplified using the primer pair ckatE-F and ckatE-R (Table 1). The amplified sequence included the putative promoter sequence of the *katE* gene, previously predicted with SoftBerry (www.softberry.com). The amplified DNA fragment was then cloned into the broad-host-range vector pBBR1MCS-5 [25] to generate the recombinant plasmid pBBR1/*katE*. This plasmid was transferred into the Xac*katE* mutant strain by conjugation, rendering strain cXac*katE* (Table 1).

### Plant material and plant inoculations

Orange (*Citrus sinensis* cv. Valencia) was used as the host plant for Xac. All plants were grown in a growth chamber in incandescent light at 25°C with a photoperiod of 16 h. Overnight cultures of Xac WT, Xac*katE* and cXac*katE* were diluted in 10 mM MgCl_2_ to a final concentration of 10^5^ CFU ml^−1^. For disease symptoms assays, bacterial suspensions were infiltrated into leaves with needleless syringes. *In planta* growth assays were performed by grinding 0.8 cm diameter leaf discs from infiltrated leaves in 100 µl of 10 mM MgCl_2_, followed by serial dilutions and plating onto SB-agar plates. Colonies were counted after 48 h incubation at 28°C.

## Results

### Catalase activity pattern is regulated in different growth stages

**Figure 1:**
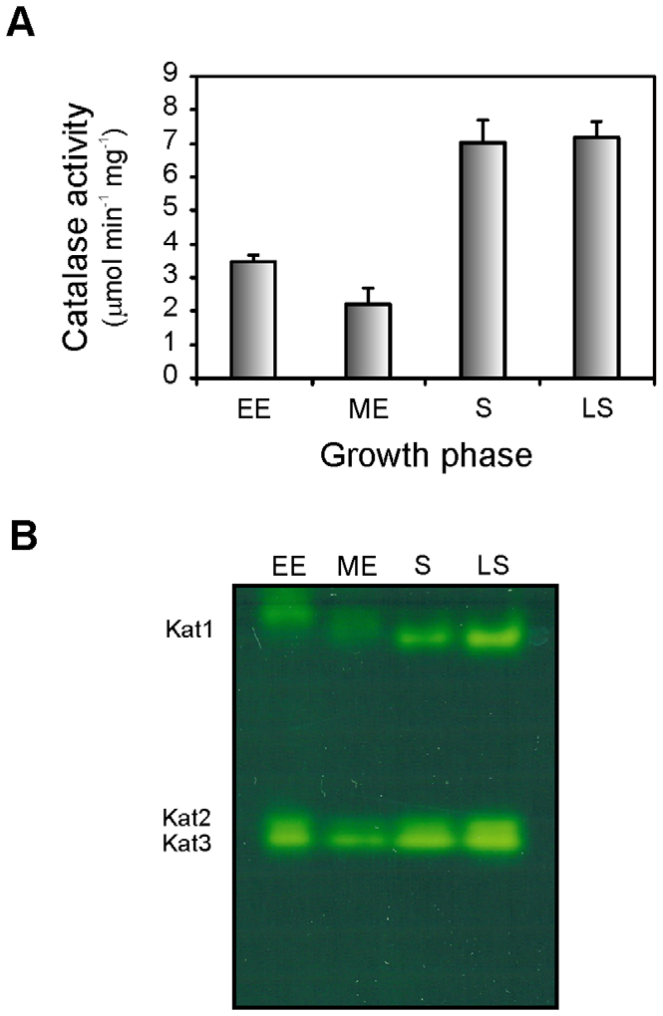
Catalase activity in Xac as influenced by the growth phase. (A) Xac cultures were grown aerobically in SB medium to early exponential (EE, 4 h), mid-exponential (ME, 8 h), stationary (S, 24 h) and late stationary (LS, 48 h) phases, and soluble extracts were prepared as described in *Materials and Methods*. Total catalase activity was assayed as described by Beers and Sizer [18] with 10 mM H_2_O_2_ at 25°C. (B) Equal amounts of protein (25 µg) were separated by 8% non-denaturing PAGE and stained for catalase activity by the method of Scandalios [19]. A simultaneously run Coomassie-stained gel (not shown) indicated equal protein loadings between samples. The positions of the electrophoretically discernible catalase species Kat1, Kat2, and Kat3 are indicated.

We investigated the growth phase-dependent pattern of catalase activity in Xac by conducting activity assays on soluble extracts from cultures at different growth stages. A typical growth curve of Xac in SB medium is depicted in Figure S1. As shown in Figure 1A, the highest levels of catalase activity were observed in the stationary and late stationary phases (with similar values of ∼7 µmol min^−1^ mg^−1^), being approximately 2.5-fold higher than those determined for the cultures in exponential growth.

On the other hand, equal amounts of the bacterial extracts were separated by 8% non-denaturing PAGE and subsequently stained for catalase activity (Figure 1B). Three distinct catalase bands were detected throughout all stages of growth: a slow-migrating catalase denoted Kat1, and two bands with similar electrophoretic mobilities that were named Kat2 and Kat3. The activity level of Kat1 increases significantly in the stationary phase of growth, whereas the levels of Kat2 and Kat3 decline in the mid-exponential phase and increase again as cells enter and remain in the stationary phase.

The intensities of the bands detected in the activity gel were measured using Gel-Pro Analyzer Software 3.1 (Media Cybernetics) and the total optical density for each growth stage was calculated. The pattern obtained was consistent with the activity measurements depicted in Figure 1A (data not shown).

### Xac in the stationary phase of growth is more resistant to hydrogen peroxide

**Figure 2:**
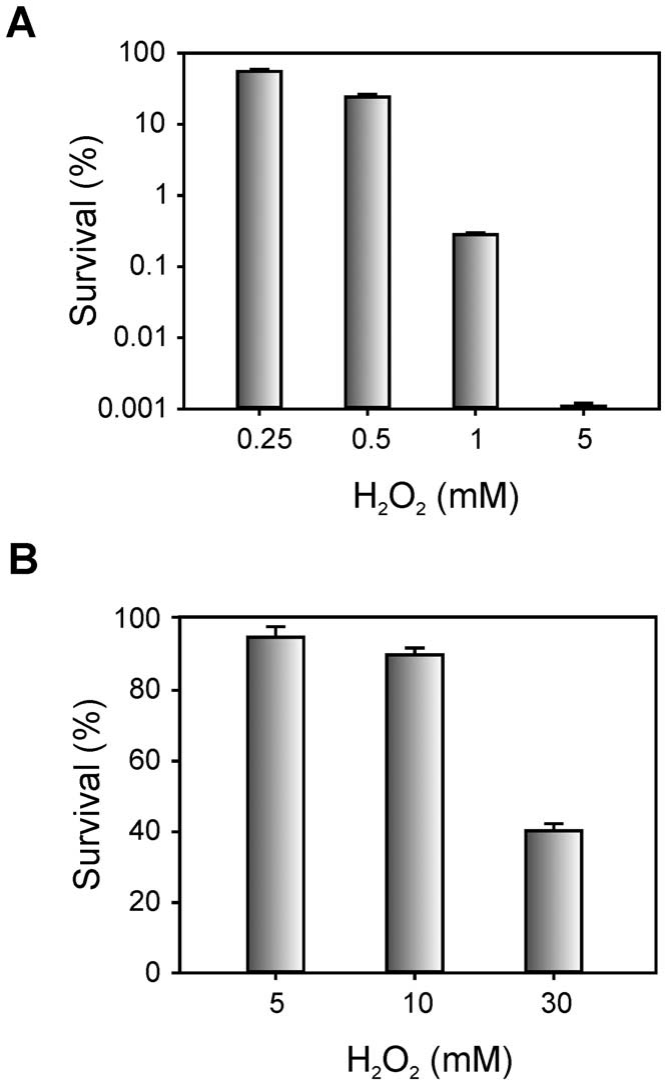
Hydrogen peroxide resistance of Xac cultures in different growth stages. Cells in early exponential (A) or stationary (B) phase of growth were exposed to the indicated concentrations of H_2_O_2_ for 15 min. The number of CFU was determined for each culture before and after the peroxide treatment by plating of appropriate dilutions. The percentage of survival is defined as the number of CFU after treatment divided by the number of CFU prior to treatment ×100. Data are expressed as the mean ± standard deviation of three independent experiments.

In order to investigate whether the elevated catalase activity observed in the stationary phase provides Xac cultures with enhanced resistance to oxidative stress, studies of bacterial survival in the presence of H_2_O_2_ were performed (Figure 2). In early exponential phase, Xac was very sensitive to hydrogen peroxide treatment, with only 0.28% survival following addition of 1 mM H_2_O_2_ and almost no detectable CFU following treatment with 5 mM H_2_O_2_. On the other hand, Xac in stationary phase of growth was significantly more resistant to the oxidative stress treatment, with 95% survival following the addition of 5 mM H_2_O_2_ and 40% survival following treatment with 30 mM H_2_O_2_.

### Identification of the catalase isoforms expressed in the different growth stages

Analysis of the Xac genome sequence revealed the presence of four putative catalase genes designated as *katE*, *srpA*, *catB* and *katG* [14], [15]. Comparative sequence analysis of the encoded proteins were performed by using ClustalX [26] and described in Supporting Information S1 and Figures S2, S3, S4 and S5.

**Figure 3:**
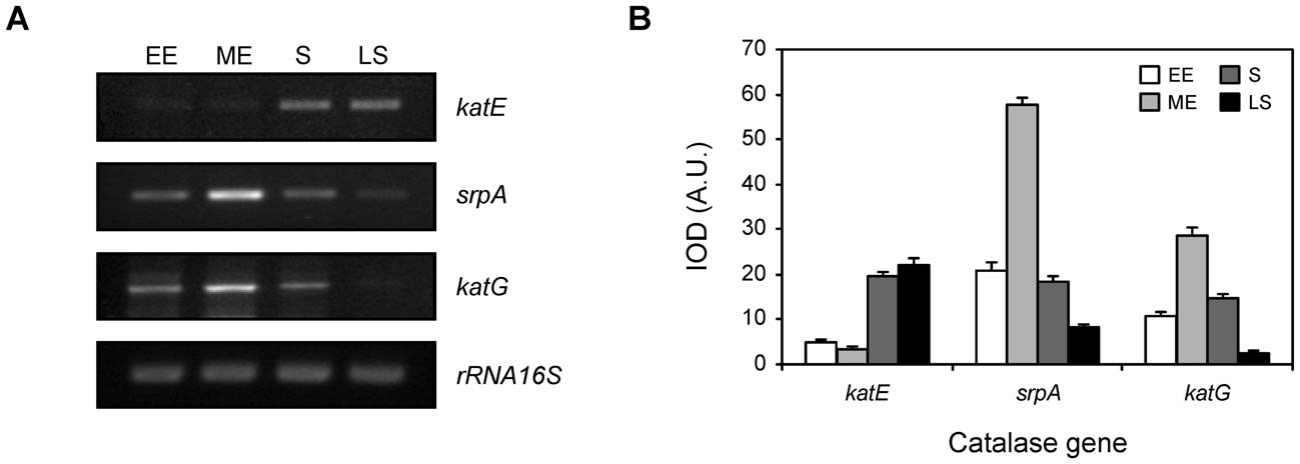
Expression analysis of Xac catalase genes as a function of the growth phase. (A) Amplified products of the *katE*, *srpA* and *katG* genes by semi-quantitative RT-PCR using RNA preparations from Xac cultures grown in SB medium to early exponential (EE, 4 h), mid-exponential (ME, 8 h), stationary (S, 24 h) and late stationary (LS, 48 h) phases. 16S rRNA was used as a loading control and to quantitate the amount of RNA in RT-PCRs. (B) Expression profiles obtained by densitometric quantification of band intensities. Experiments were performed in triplicate with similar results; error bars indicate ±1 standard deviation of the mean. IOD, integrated optical density; A.U., arbitrary units.

In order to investigate the expression profiles of the Xac catalase genes during growth, we performed semi-quantitative RT-PCR reactions using specific primers designed from the reported gene sequences (Table 1, Figure 3). As a control for constitutive bacterial expression a fragment of 16S rRNA was simultaneously amplified. Expression of *katE* was hardly detectable at the early and mid-exponential phases of growth, subsequently increasing to reach 5-fold higher levels during the stationary phase. On the other hand, expression of *srpA* and *katG* genes was detected throughout all stages of growth, reaching maximal levels in the mid-exponential phase and decreasing gradually towards the stationary phase. The mRNA levels of *katG* were almost undetectable in the late stationary phase. The *catB* gene was not included in the figure since no product was observed in the RT-PCR reactions under the conditions tested. To ascertain the absence of contaminating DNA in bacterial RNA samples control PCR reactions where RT was omitted were carried out in parallel for all samples (data not shown).

### Xac adaptive response to hydrogen peroxide

The adaptive response to oxidative stress agents is a well-characterized phenomenon observed in many bacteria, in which the exposure to sub-lethal levels of an oxidant leads to the induction of genes involved in the bacterial stress response, ultimately conferring resistance to lethal levels of the same agent or even unrelated compounds (cross protection) [27]. The ability to develop an adaptive response to hydrogen peroxide was investigated in Xac by determining the catalase activity in early exponential cultures incubated with sub-lethal concentrations of H_2_O_2_ (10, 30 and 100 µM) for 60 min. As shown in Table 2, a 2-fold induction of catalase activity was observed in cultures treated with 100 µM H_2_O_2_ with respect to the untreated control cells.

**Table 2:** Induction of catalase activity in response to sub-lethal levels of hydrogen peroxide^a^.

Culture	Catalase activity	Induction
	(µmol min^−1^ mg^−1^ protein)	(fold)
Uninduced	3.7±0.3	-
Induced by H_2_O_2_		
10 µM	4.5±0.2	1.2
30 µM	5.7±0.2	1.5
100 µM	7.6±0.4	2.0

**a.** Xac cells were grown in SB medium to early exponential phase and exposed to the indicated concentrations of H_2_O_2_ for 1 hour. Catalase activities in soluble cell extracts were measured as described in *Materials and Methods*.

Data represent mean ± standard deviation of three independent experiments.

**Figure 4:**
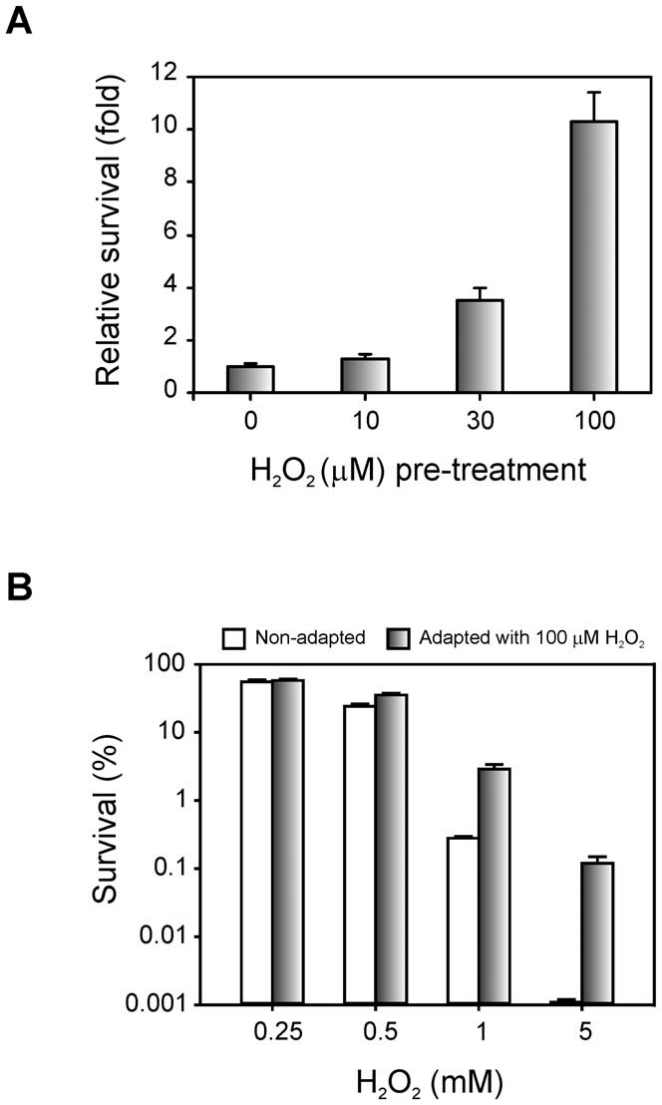
Adaptive response of Xac to hydrogen peroxide treatment. (A) Exponential phase cultures were adapted with the indicated concentrations of H_2_O_2_ for 60 min and then exposed to 1 mM H_2_O_2_ for 15 min. The number of CFU was determined for each culture before and after the treatment with 1 mM H_2_O_2_ by plating of appropriate dilutions. The related survival is defined as the percentage of survival of the pre-adapted culture divided by the percentage of survival of the untreated control. (B) Exponential phase cultures were pre-adapted with 100 µM H_2_O_2_ for 60 min. The number of CFU was determined for the preadapted cultures and for the unadapted controls and then H_2_O_2_ was added to the final concentrations indicated, followed by an incubation of 15 min. The percentage of survival was calculated as the number of CFU after treatment divided by the number of CFU prior to treatment ×100. Experiments were performed in triplicate; error bars indicate ±1 standard deviation of the mean.

Based on this observation, the resistance of bacterial cells pre-adapted with sub-lethal levels of H_2_O_2_ to a lethal dose of the same agent was assessed. Cultures pre-treated with 10, 30 and 100 µM H_2_O_2_ were subsequently challenged with a killing concentration of H_2_O_2_ (1 mM, see Figure 2A) and the percentages of survival were determined (Figure 4A). Interestingly, a dose dependent response was observed with these H_2_O_2_ concentrations, with a 10-fold increase in resistance after pre-adaptation with 100 µM H_2_O_2_. Moreover, Xac cultures pre-treated with 100 µM H_2_O_2_ for 1 hour were then incubated with 0.25, 0.5, 1 and 5 mM H_2_O_2_ for 15 min (Figure 4B). We found that pre-adapted cells were more resistant than the control cells to all H_2_O_2_ concentrations tested, the difference of survival being more pronounced as the H_2_O_2_ levels increases. After challenge with 5 mM H_2_O_2_ survival of the pre-adapted culture was 100-fold higher than that of the untreated control.

### A medium that mimics the environment of plant intercellular spaces modifies Xac catalase expression pattern

As an initial approach to evaluate the involvement of catalases during plant-pathogen interactions we determined the levels of these enzymes in early exponential and stationary phase cultures grown in NB, a rich standard medium, and in XVM2, a nutrient poor medium that simulates conditions in the apoplastic space of plants, which induces the bacterial *hrp* (for *h*ypersensitive *r*esponse and *p*athogenicity) gene cluster [23]. Typical growth curves of Xac in these media are depicted in Figure S6. As shown in Table 3, cells grown in XVM2 exhibited ∼2-fold higher catalase activity levels than cells grown in the standard medium, suggesting a possible induction of these enzymes in the environment found in the intercellular spaces of plant tissues. Moreover, Xac cultures grown in XVM2 were considerably more resistant to killing when exposed to H_2_O_2_ than those grown in NB (Table 3).

**Table 3:** Increase in catalase activity and hydrogen peroxide resistance of Xac cells in a plant-like medium.

Medium	Catalase activity^a^	% Survival^b^
	(µmol min^−1^ mg^−1^ protein)	H_2_O_2_
*Early exponential phase*		
NB	3.2±0.2	0.25±0.07
XVM2	7.5±0.3	3.4±0.1
*Stationary phase*		
NB	7.2±0.4	38±2
XVM2	16.3±0.6	98±5

**a.** Xac cells were grown in the indicated media to early exponential or stationary phase and then harvested. Catalase activities in soluble cell extracts were measured as described in *Materials and Methods*.

**b.** Early exponential and stationary phase cultures were exposed to 1 mM and 30 mM H_2_O_2_ respectively, for 15 min. The percentage of survival is defined as the number of CFU after treatment divided by the number of CFU prior to treatment ×100.

Data represent mean ± standard deviation of three independent experiments.

**Figure 5:**
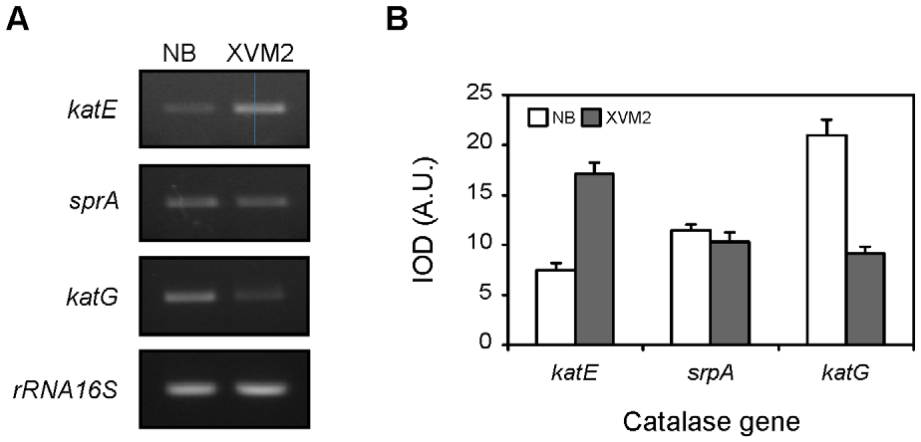
Expression of Xac catalase genes in the plant-mimicking XVM2 medium. (A) Amplified products of the catalase genes by semi-quantitative RT-PCR using RNA preparations from early exponential Xac cultures grown in NB and in XVM2. As a control for constitutive bacterial expression a fragment of 16S rRNA was simultaneously amplified. (B) Expression profiles obtained by densitometric quantification of band intensities. Experiments were performed in triplicate with similar results; error bars indicate ±1 standard deviation of the mean. IOD, integrated optical density; A.U., arbitrary units.

**Figure 6:**
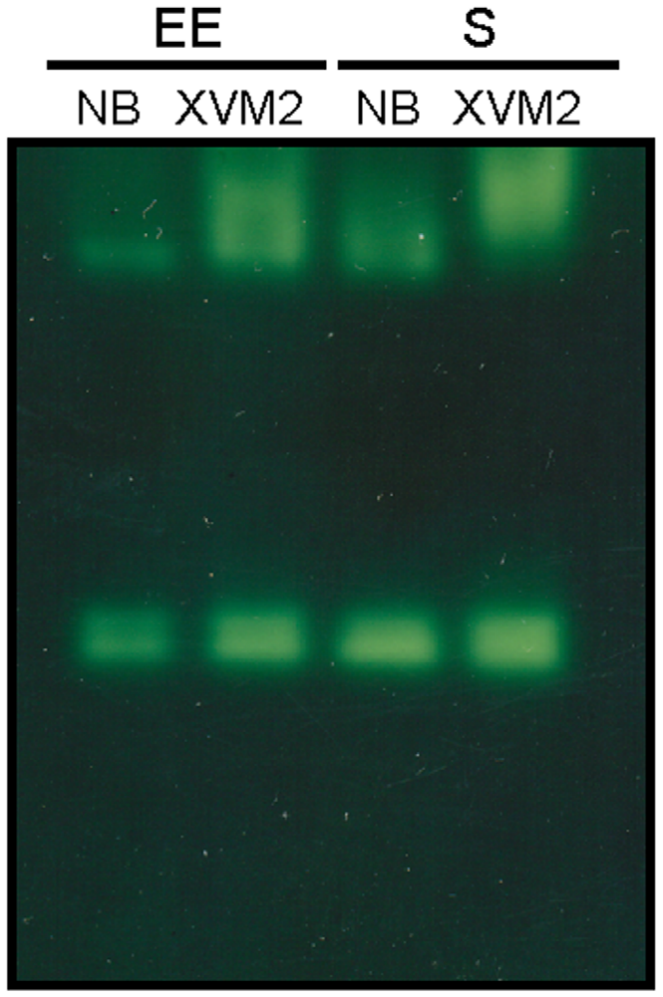
Detection of catalase activity in Xac cultures grown in NB and XVM2 media. Xac cultures were grown aerobically in NB and XVM2 media to early exponential (EE, 7 h), and stationary (S, 16 h) phases, and soluble extracts were prepared as described in *Materials and Methods*. Equal amounts of protein (40 µg) were separated by 8% non-denaturing PAGE and stained for catalase activity by the method of Scandalios [19]. A simultaneously run Coomassie-stained gel (not shown) indicated equal protein loadings between samples.

To address the question if there is a transcriptional induction of any of the catalase genes in the XVM2 medium we performed semiquantitative RT-PCR analysis with early exponential phase cultures. Interestingly, different expression patterns were observed depending on the growth conditions (Figure 5). While mRNA levels of *srpA* were similar in both media, expression of *katE* was ∼2.3-fold higher in XVM2 than in NB, whereas the levels of *katG* exhibited a reduction of similar magnitude in the minimal medium. Analysis of the catalase activity in native polyacrylamide gels revealed that the intensity of the slow-migrating band (Kat1, Figure 1B) was markedly raised in the plant-like medium, both in exponential and stationary growth phases (Figure 6).

### Characterization of a Xac*katE* mutant strain

Having established that *katE* is transcriptionally induced in Xac during the stationary phase of growth and in the apoplastic space mimicking XVM2 medium, a Xac*katE* mutant strain was then generated by insertional mutagenesis (see *Materials and Methods*) and genetically verified by PCR analysis (data not shown).

**Figure 7:**
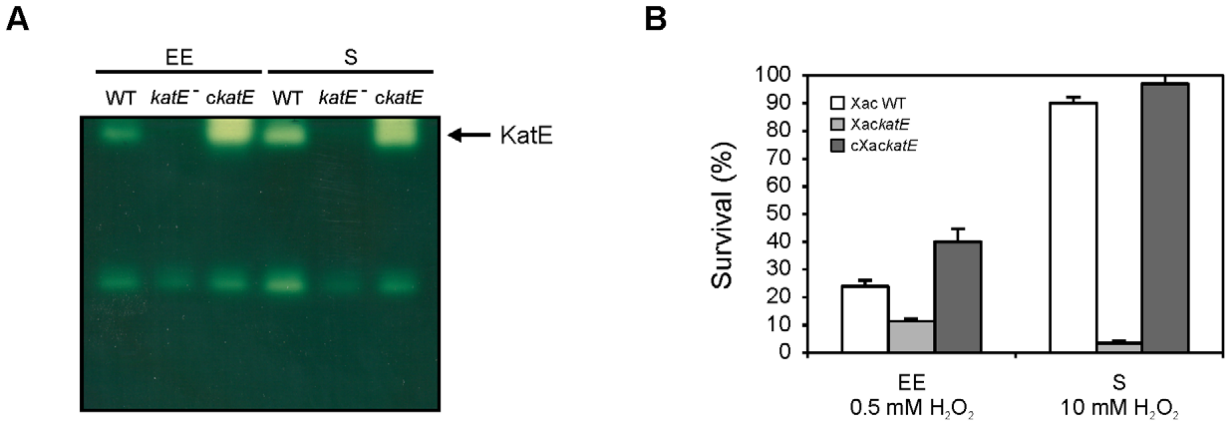
Catalase activity and hydrogen peroxide resistance in the Xac*katE* mutant. (A) Xac wild-type (WT), Xac*katE* (*katE^−^*) and cXac*katE* (c*katE*) strains were grown aerobically in SB medium to early exponential (EE, 4 h) and stationary (S, 24 h) phases, and soluble extracts were prepared as described in *Materials and Methods*. Equal amounts of protein (25 µg) were separated by 8% non-denaturing PAGE and stained for catalase activity by the method of Scandalios [19]. (B) Cells in early exponential (EE) or stationary (S) phase of growth were exposed to the indicated concentrations of H_2_O_2_ for 15 min. The number of CFU was determined for each culture before and after the peroxide treatment by plating of appropriate dilutions. The percentage of survival is defined as the number of CFU after treatment divided by the number of CFU prior to treatment ×100. Data are expressed as the mean ± standard deviation of three independent experiments.

In order to assess the effect of *katE* disruption on the catalase pattern, soluble extracts from the parental (WT) and mutant (*katE*) strains in early exponential and stationary phases of growth were analyzed by native gel electrophoresis and catalase staining. As shown in Figure 7A, the upper band observed in the wild-type strain was completely absent in the *katE* mutant, indicating that this band corresponds to KatE. A complementation assay was also carried out to validate the *katE* phenotype. This was done by cloning the *katE* gene under the control of its own promoter sequence in a pBBR1MCS-5 vector [25], which was then conjugated into the *katE* mutant. The upper catalase band was recovered in the resulting cXac*katE* strain, corroborating the identity of this catalase (Figure 7A). The intensity of this band was higher than the observed in the wild-type cells, probably due to the low but still multiple copy number of the pBBR1/*katE* vector in Xac cells.

The growth phase-dependent pattern of catalase activity in the Xac*katE* strain was then investigated by conducting assays on soluble extracts from cultures in different stages of growth. In contrast to wild-type bacteria, no induction was observed in stationary phase cultures of the Xac*katE* mutant, with a constant average value of ∼1.6 µmol min^−1^ mg^−1^ throughout all the bacterial growth cycle. On the other hand, the cXac*katE* strain exhibited the same pattern of wild-type cells but with higher activity values.

Furthermore, the mutant strain was more sensitive to hydrogen peroxide treatment in both early exponential and stationary growth phases (Figure 7B).

The Xac*katE* adaptive response to hydrogen peroxide was also analyzed by determining the catalase activity in early exponential cultures incubated with sub-lethal concentrations of the oxidant. As was previously demonstrated for wild-type cells, a ∼2-fold induction of catalase activity was also observed in Xac*katE* cells treated with 100 µM H_2_O_2_ (Table 4), suggesting that KatE is not responsible for this response.

**Table 4:** Induction of catalase activity in the Xac*katE* mutant in response to sub-lethal levels of hydrogen peroxide^a^.

Culture	Catalase activity	Induction
	(µmol min^−1^ mg^−1^ protein)	(fold)
Uninduced	1.7±0.2	-
Induced by H_2_O_2_		
30 µM	2.7±0.2	1.6
100 µM	3.2±0.3	1.9

**a.** Xac*katE* cells were grown in SB medium to early exponential phase and exposed to the indicated concentrations of H_2_O_2_ for 1 hour. Catalase activities in soluble cell extracts were measured as described in *Materials and Methods*.

Data represent mean ± standard deviation of three independent experiments.

### Interaction of the Xac*katE* mutant with host plants

**Figure 8:**
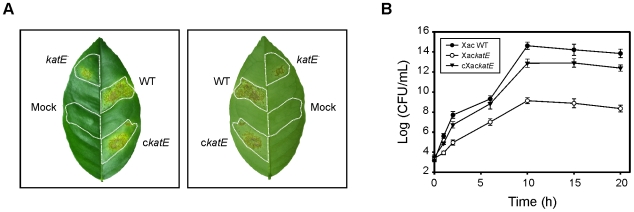
Effect of *katE* disruption on pathogenicity. Xac WT (WT), Xac*katE* (*katE* ^−^) and cXac*katE* (c*katE*) cells were inoculated at 10^5^ CFU ml^−1^ in 10 mM MgCl_2_ into the intercellular spaces of fully expanded orange leaves. (A) A representative leaf 20 days after inoculation is shown. Left panel, adaxial side; right panel, abaxial side. Dashed lines indicate the infiltrated area. (B) Bacterial growth of Xac cells in orange leaves. Values represent means of three independent samples; error bars represent standard deviations.

In order to assess the physiological role of KatE during the infection process, the mutant strain was tested for its ability to trigger disease in citrus leaves. Both wild-type bacteria and Xac*katE* produced typical canker lesions upon infiltration at a concentration of 10^5^ CFU ml^−1^, with no differences in the time of appearance of the first symptoms (water soaking). However, the magnitude of the lesions and the number of cankers were significantly diminished in the mutant strain compared to wild-type bacteria, even though the infiltration areas and the bacterial densities were equivalent for both strains. On the other hand, infiltration with the cXac*katE* strain caused the same symptoms and a similar percentage of necrotic area than wild-type cells (Figure 8A).

The degree of virulence of the different strains was also evaluated by conducting bacterial growth curves *in planta*. As shown in Figure 8B, the magnitudes of leaf injuries correlated with the bacterial growths inside the host. The bacterial number of Xac*katE* recovered from the infected leaves was fewer than that of the wild-type strain at each time analyzed. On the other hand, although complementation restored the bacteria to full virulence on citrus leaves, cXac*katE* growth on leaves did not reach the values of the wild-type.

Furthermore, in order to rule out the possibility that the lower infectivity of Xac*katE* arise because mutant cells had already been injured during the culture period, infection experiments were conducted with exponentially growing cultures, in which KatE would not be even induced. The results obtained in these inoculations were in agreement with those previously described, indicating that the deficiency suffered by the mutant strain arise during the plant infection (data not shown).

## Discussion

*X. axonopodis* pv. *citri* holds a strictly aerobic life style and this physiology can lead to the intracellular generation of oxidative stress during normal respiration on molecular oxygen. As a pathogenic microorganism, it encounters a great deal of oxidative stress during the infection process as well. To prevent the accumulation of ROS generated during aerobic respiration or plant interactions, Xac should employ versatile antioxidant defense enzymes, including catalases. The genome of Xac has been completely sequenced, revealing the presence of four putative catalases, four SODs and the OxyR and SoxR sensors [14], [15]. The elevated number of genes encoding for antioxidant enzymes in this bacterium provides an indication of the overall relevance of the antioxidant systems for its survival. In this study we focused on the analysis of Xac catalases and their expression patterns in order to elucidate the physiological roles that catalases play in this microorganism.

The pattern of catalase activity was found to be growth phase-regulated in Xac, with the highest levels detected during the stationary phase (Figure 1). This result was unexpected because previous reports in other *Xanthomonas* species showed that maximum levels of enzyme activity were attained as the cultures were emerging from the lag phase and subsequently declined as growth proceeded [28]–[30]. On the other hand, growth into stationary phase has been largely documented as one of the main factors influencing catalase levels in a majority of bacteria, as these enzymes would serve a protective role against peroxide during periods of low metabolic activity [7], [31], [32]. According to our results this may be the case for Xac, possibly suggesting different mechanisms of catalase regulation between species of the *Xanthomonas* genus. In addition, we showed that resistance levels to H_2_O_2_ treatment also varies significantly in Xac depending on the growth stage, with stationary phase cells being capable of tolerating up to 30-fold higher concentrations of the oxidant than exponentially growing cells (Figure 2).

The decrease in the activities of oxidant-scavenging enzymes, such as catalase and SOD, observed in other *Xanthomonas* species during the stationary phase of growth has lead to the proposal that the mechanisms responsible for stationary-phase resistance to oxidants would be independent of the levels of scavenging enzymes [29]. In contrast, our results revealed that Xac resistance to H_2_O_2_ during the bacterial growth cycle increases in parallel with the expression of catalase-specific activity (Figures 1 and 2), suggesting that the mechanism of resistance to oxidants in this bacterium differs at least partially from those reported for other species of the *Xanthomonas* genus.

We also demonstrated that Xac catalase activity is regulated at isozymes level. At all stages of Xac growth we were able to detect three bands with catalase activity in non-denaturing gels (Figure 1B), which exhibited differential patterns of expression along the bacterial growth cycle, being the upper band significantly induced during the stationary phase.

Additionally, we analyzed the expression of the complete set of Xac catalase genes (*katE, katG, catB* and *srpA*) along the bacterial growth cycle by RT-PCR, showing that the *katE* gene was strongly induced during the stationary phase, while *katG* and *srpA* exhibited a peak level of expression during the mid-exponential phase (Figure 3). On the other hand, transcription of the *catB* gene was not detected under the conditions tested, suggesting that the gene may be cryptic, that is, present but not expressed in Xac. This may be attribuited to the fact that there are two overlapping gene fragments annotated as *catB* in the Xac genome sequence (XAC4029 and XAC4030), which encode for this putative monofunctional catalase in different open reading frames [14], [15]. However, the possibility that *catB* is expressed in Xac under specific growth conditions not assayed for in this study can not be ruled out.

Interestingly, transcript levels of *katG* and *srpA* in the mid-exponential phase were equal to or even higher than the total transcript levels observed during the stationary or late-stationary phases (Figure 3). However, total catalase activity detected in the stationary phase was significantly higher than that of the mid-exponential phase (Figure 1A). We speculate that the apparent discrepancy between RNA levels and catalase activities may be a consequence of the rapid rate of bacterial duplication during the exponential phase of the growth cycle, which may cause the limitation of some component (e.g., heme and/or iron) essential for the proper assembly/activity of the enzyme. A potential mechanism of post-transcriptional regulation could also be involved in the control of the catalase expression. Further investigation would be necessary to probe this contention.

We have also demonstrated that interruption of the *katE* gene has a marked effect on catalase activity in growth-arrested cells. The Xac*katE* mutant strain exhibited a constant low level of activity throughout all the bacterial growth cycle and lower resistance to H_2_O_2_ than wild-type cells, the difference of survival being more pronounced during the stationary phase (Figure 7B). These findings support the notion that KatE is the isozyme responsible for the increase of catalase activity previously observed for the wild-type strain during the stationary phase. Furthermore, this catalase accounts for a considerable part of the overall hydrogen peroxide resistance in Xac. On the other hand, the catalase content of the Xac*katE* mutant on non-denaturing gels revealed the absence of the upper activity band of the wild-type strain (Kat1 in Figure 1B), which allowed us to conclude that this band corresponds to the KatE isozyme (Figure 7A). Moreover, the absence of this band in both phases of growth supports the notion that the apparently different mobility observed between lanes in Figure 1 was only an artifactual effect of the electrophoretic run. In addition, a decrease in the intensities of the bands with higher electrophoretic mobilities (Kat2 and Kat3) was also observed in these gels, suggesting that the loss of KatE influences the expression of the other catalase isoforms.

The adaptive response to oxidative agents has been previously proposed to play a fundamental role in plant-pathogen interactions, allowing bacteria to withstand increased oxidative stress conditions [27]. We then became interested in the adaptive response of Xac to H_2_O_2_, the major component of the plant oxidative burst [13]. Our results demonstrate that Xac also develops an adaptive response to H_2_O_2_, and the level of induced protection correlates with the bacterial ability to induce catalase activity during the pre-adaptation treatment (Table 2). Since the Xac*katE* mutant exhibited the same catalase activity induction than wild-type cells after the oxidative treatment, we suggest that KatE would not be involved in the adaptive response of Xac.

Adequacy of the antioxidant system may be critical for Xac interaction with citrus plants, in order to minimize oxidative stress and establish infection. We observed that the total catalase activity and the resistance to H_2_O_2_ were significantly higher in the apoplastic space mimicking XVM2 medium than in a rich medium (NB) (Table 3). The expression analysis of the different catalase genes indicated that the monofunctional catalase *katE* was strongly induced in XVM2 (Figure 5). Consistent with this, analysis of the upstream sequence of the *katE* gene revealed the presence of an imperfect PIP box (TTCGCN_14_TTCGT) located 1 bp downstream of the predicted −10 promoter sequence. This conserved plant-inducible promoter sequence motif has been suggested to be associated with the regulation of genes induced *in planta* and also in the XVM2 medium [33]. Our results indicate that Xac catalase expression pattern is modified in response to any stimuli associated with the plant or the microenvironment it provides. It is still not clear if the increase in catalase activity observed in XVM2 was only a result of higher expression of the *katE* gene (due to possible differences in the catalytic properties with respect to KatG), or enhanced enzyme activity due to post-transcriptional regulation, or both. Nevertheless, the induction of catalase activity in response to the plant environment may serve a protective role against exposure to H_2_O_2_ in the early stages of plant infection.

Furthermore, the virulence of the Xac*katE* mutant was considerably attenuated during the compatible interaction with citrus plants, given that the magnitude of the damaged tissue and the number of canker lesions were noticeably reduced compared to the wild-type strain. The phenotypic differences observed between both strains were consistent with the bacterial growth curves *in planta* (Figure 8). The wild-type virulence was recovered in a complemented strain (cXac*katE*) allowing us to conclude that the phenotypes observed are indeed caused by the loss of KatE function. Our results indicate that catalase KatE has an important function in the colonization and survival of Xac in the host tissue. Accordingly, in *X. campestris* pv. *campestris* was recently shown that a mutant in KatG, the bifunctional catalase-peroxidase of this bacterium, was unable to infect radish (*Raphanus sativus*) leaves and cause disease [34]. The impaired ability of the Xac*katE* mutant to infect citrus leaves provides the first genetic evidence to support a monofunctional catalase as a virulence factor in Xac, further indicating that the oxidative burst may play a significant role in pathogen growth restriction during the infection process.

Our results collectively suggest that in the apoplast environment Xac may be more resistant to H_2_O_2_ as a consequence of KatE induction. Our future aims are to elucidate the regulatory pathways that orchestrate the Xac oxidative stress response during the first stages of plant infection.

## Supporting Information

Supporting Information S1 The monofunctional catalase KatE of *Xanthomonas axonopodis* pv. *citri* is required for full virulence in citrus plants.
(0.06 MB DOC)

Figure S1 Growth curve of Xac in SB medium. Xac culture was cultivated aerobically in SB medium at 28°C with shaking at 200 rpm. Aliquots were taken at the indicated times and measured for both optical density at 600 nm (OD_600_, open circles) and colony-forming capacity on SB-agar medium (closed circles).
(2.04 MB TIF)

Figure S2 Multiple alignment of the deduced amino acid sequence of Xac KatE (XacE) with catalases KatE of *X. campestris* pv. *phaseoli* (XcpE) and HPII of *E. coli* (EcoII), performed by using ClustalX [26]. An asterisk indicates complete residue conservation, a colon indicates strong group conservation, a period indicates weak group conservation, and a blank space indicates no conservation of residues.
(0.47 MB TIF)

Figure S3 Multiple alignment of the deduced amino acid sequence of Xac SrpA (XacA) with catalases from *X. campestris* pv. *vesicatoria* (Xcv), *X. oryzae* pv. *oryzae* (Xoo), *P. syringae* (Psy) and *P. aeruginosa* (Pae), performed by using ClustalX [26]. An asterisk indicates complete residue conservation, a colon indicates strong group conservation, a period indicates weak group conservation, and a blank space indicates no conservation of residues.
(0.45 MB TIF)

Figure S4 Alignment of the deduced amino acid sequences of Xac CatB precursor (XacBp) (A) and Xac CatB (XacB) (B) with KatA of *X. campestris* pv. *phaseoli* (XcpA), performed by using ClustalX [26]. An asterisk indicates complete residue conservation, a colon indicates strong group conservation, a period indicates weak group conservation, and a blank space indicates no conservation of residues.
(0.47 MB TIF)

Figure S5 Multiple alignment of the deduced amino acid sequence of Xac KatG (XacG) with catalases from *X. campestris* pv. *vesicatoria* (Xcv) and *X. campestris* pv. *campestris* (Xcc), and the bifunctional HPI of *E. coli* (EcoI), performed by using ClustalX [26]. An asterisk indicates complete residue conservation, a colon indicates strong group conservation, a period indicates weak group conservation, and a blank space indicates no conservation of residues.
(0.60 MB TIF)

Figure S6 Growth curves of Xac in NB and XVM2 media. Xac cultures were cultivated aerobically in these media at 28°C with shaking at 200 rpm. Aliquots were taken at the indicated times and measured for optical density at 600 nm (OD_600_).
(1.88 MB TIF)
