# Supplementary material for: The Monofunctional Catalase KatE of Xanthomonas axonopodis pv. citri Is Required for Full Virulence in Citrus Plants
Source: PLoS One. 2010 May 24;5(5):e10803. doi: 10.1371/journal.pone.0010803 (PMC2875408; doi:10.1371/journal.pone.0010803)
Supplement: Supporting Information S1 — The monofunctional catalase KatE of Xanthomonas axonopodis pv. citri is required for full virulence in citrus plants. (0.06 MB DOC) [file pone.0010803.s001.doc]

**The monofunctional catalase KatE of *Xanthomonas axonopodis* pv. *citri* is required for full virulence in citrus plants**

María Laura Tondo, Silvana Petrocelli, Jorgelina Ottado and Elena G. Orellano ^¶^

Supporting Information S1

# Results

# *Primary sequence analysis of Xac catalases*

Four putative catalase genes have been identified in the Xac genome sequence [1,2]. Xac *katE* gene (XAC1211) encodes for a predicted 703-aa monofunctional catalase with an expected molecular mass of 76.6 kDa. GenBank comparisons of Xac KatE revealed 97% amino acid sequence identity with KatE of the related species *X*. *campestris* pv. *phaseoli*, the monofunctional isozyme that is growth-phase regulated in this bacterium [3] (Figure S2). Sequence homology has also been found between Xac KatE and *E. coli* HPII (KatE) [4], the monofunctional catalase transcriptionally induced in *E. coli* during the stationary phase of growth.

A second gene encoding a putative 39-kDa monofunctional catalase has been annotated in the Xac genome sequence as *srpA* (XAC3990). The comparative sequence analysis of the protein encoded by Xac *srpA* showed high homology to catalases of the related species and plant pathogens *X. campestris* pv. *vesicatoria* (97% identity) [5] and *X. oryzae* pv. *oryzae* (96% identity) [6], which have not been characterized so far (Figure S3). Amino acid sequence identity was also observed with CatF of *Pseudomonas syringae* (24% identity) [7] and KatB of *Pseudomonas aeruginosa* (23% identity) [8], the latter being involved in the bacterial response to hydrogen peroxide.

The Xac *catB* gene (XAC4029) encodes for a predicted 392-aa protein (44 kDa) identified by sequence similarity as a putative catalase precursor. Another gene, also annotated as *catB* (XAC4030) in the Xac genome sequence, overlaps the one described above but encodes a 172-aa peptide (18.5 kDa) in a different open reading frame. The predicted amino acid sequences of both genes, however, were found to be homologous to the same proteins in the GenBank search, showing high sequence identities (99 and 98%, respectively) with the monofunctional KatA of *X. campestris* pv. *phaseoli* [9] (Figure S4).

In addition to these monofunctional catalases, a bifunctional enzyme with putative catalase and peroxidase activities was found in Xac genome, encoded by the *katG* gene (XAC1301). The deduced amino acid sequence of Xac KatG (82.8 kDa) exhibited 97 and 88% identity with catalase-peroxidases of the related species *X. campestris* pv. *vesicatoria* [5] and *X.* *campestris* pv. *campestris* [10], and 62% identity with *E. coli* HPI (KatG) [11], the bifunctional catalase induced in *E. coli* during exponential growth in response to low concentrations of hydrogen peroxide (Figure S5).

**References**

1. Van Sluys MA, Monteiro-Vitorello CB, Camargo LE, Menck CF, da Silva AC et al. (2002) Comparative genomic analysis of plant-associated bacteria. Annu Rev Phytopathol 40: 169-189.

2. da Silva AC, Ferro JA, Reinach FC, Farah CS, Furlan LR et al. (2002) Comparison of the genomes of two *Xanthomonas* pathogens with differing host specificities. Nature 417: 459-463.

3. Vattanaviboon P, Mongkolsuk S (2000) Expression analysis and characterization of the mutant of a growth-phase- and starvation-regulated monofunctional catalase gene from *Xanthomonas campestris* pv. *phaseoli*. Gene 241: 259-265.

4. von O, I, Mulvey MR, Leco PA, Borys A, Loewen PC (1991) Nucleotide sequence of *Escherichia coli katE*, which encodes catalase HPII. J Bacteriol 173: 514-520.

5. Thieme F, Koebnik R, Bekel T, Berger C, Boch J et al. (2005) Insights into genome plasticity and pathogenicity of the plant pathogenic bacterium *Xanthomonas campestris* pv. *vesicatoria* revealed by the complete genome sequence. J Bacteriol 187: 7254-7266.

6. Salzberg SL, Sommer DD, Schatz MC, Phillippy AM, Rabinowicz PD et al. (2008) Genome sequence and rapid evolution of the rice pathogen *Xanthomonas oryzae* pv. *oryzae* PXO99A. BMC Genomics 9: 204.

7. Klotz MG, Kim YC, Katsuwon J, Anderson AJ (1995) Cloning, characterization and phenotypic expression in *Escherichia coli* of *catF*, which encodes the catalytic subunit of catalase isozyme CatF of *Pseudomonas syringae.* Appl Microbiol Biotechnol 43: 656-666.

8. Brown SM, Howell ML, Vasil ML, Anderson AJ, Hassett DJ (1995) Cloning and characterization of the *katB* gene of *Pseudomonas aeruginosa* encoding a hydrogen peroxide-inducible catalase: purification of KatB, cellular localization, and demonstration that it is essential for optimal resistance to hydrogen peroxide. J Bacteriol 177: 6536-6544.

9. Chauvatcharin N, Vattanaviboon P, Switala J, Loewen PC, Mongkolsuk S (2003) Cloning and characterization of *katA*, encoding the major monofunctional catalase from *Xanthomonas campestris* pv. *phaseoli* and characterization of the encoded catalase KatA. Curr Microbiol 46: 83-87.

10. Qian W, Jia Y, Ren SX, He YQ, Feng JX et al. (2005) Comparative and functional genomic analyses of the pathogenicity of phytopathogen *Xanthomonas campestris* pv. *campestris*. Genome Res 15: 757-767.

11. Triggs-Raine BL, Doble BW, Mulvey MR, Sorby PA, Loewen PC (1988) Nucleotide sequence of *katG*, encoding catalase HPI of *Escherichia coli*. J Bacteriol 170: 4415-4419.
