# Supplementary figures and images for: The Monofunctional Catalase KatE of Xanthomonas axonopodis pv. citri Is Required for Full Virulence in Citrus Plants
Source: PLoS One. 2010 May 24;5(5):e10803. doi: 10.1371/journal.pone.0010803 (PMC2875408; doi:10.1371/journal.pone.0010803)

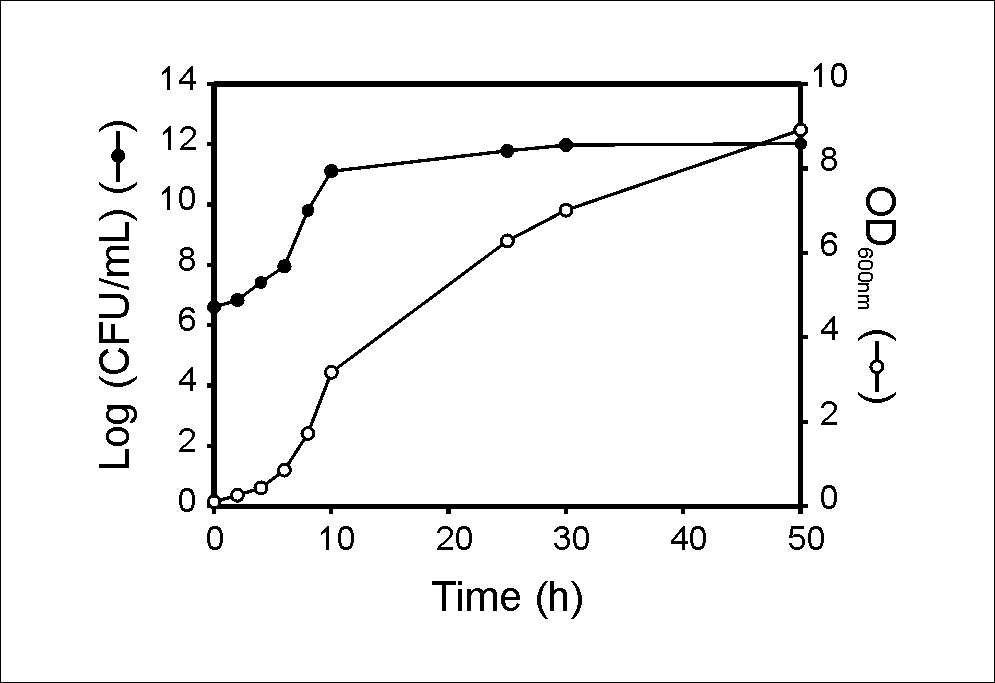

Supplement: Figure S1 — Growth curve of Xac in SB medium. Xac culture was cultivated aerobically in SB medium at 28°C with shaking at 200 rpm. Aliquots were taken at the indicated times and measured for both optical density at 600 nm (OD600, open circles) and colony-forming capacity on SB-agar medium (closed circles). (2.04 MB TIF) [file pone.0010803.s002.tif]

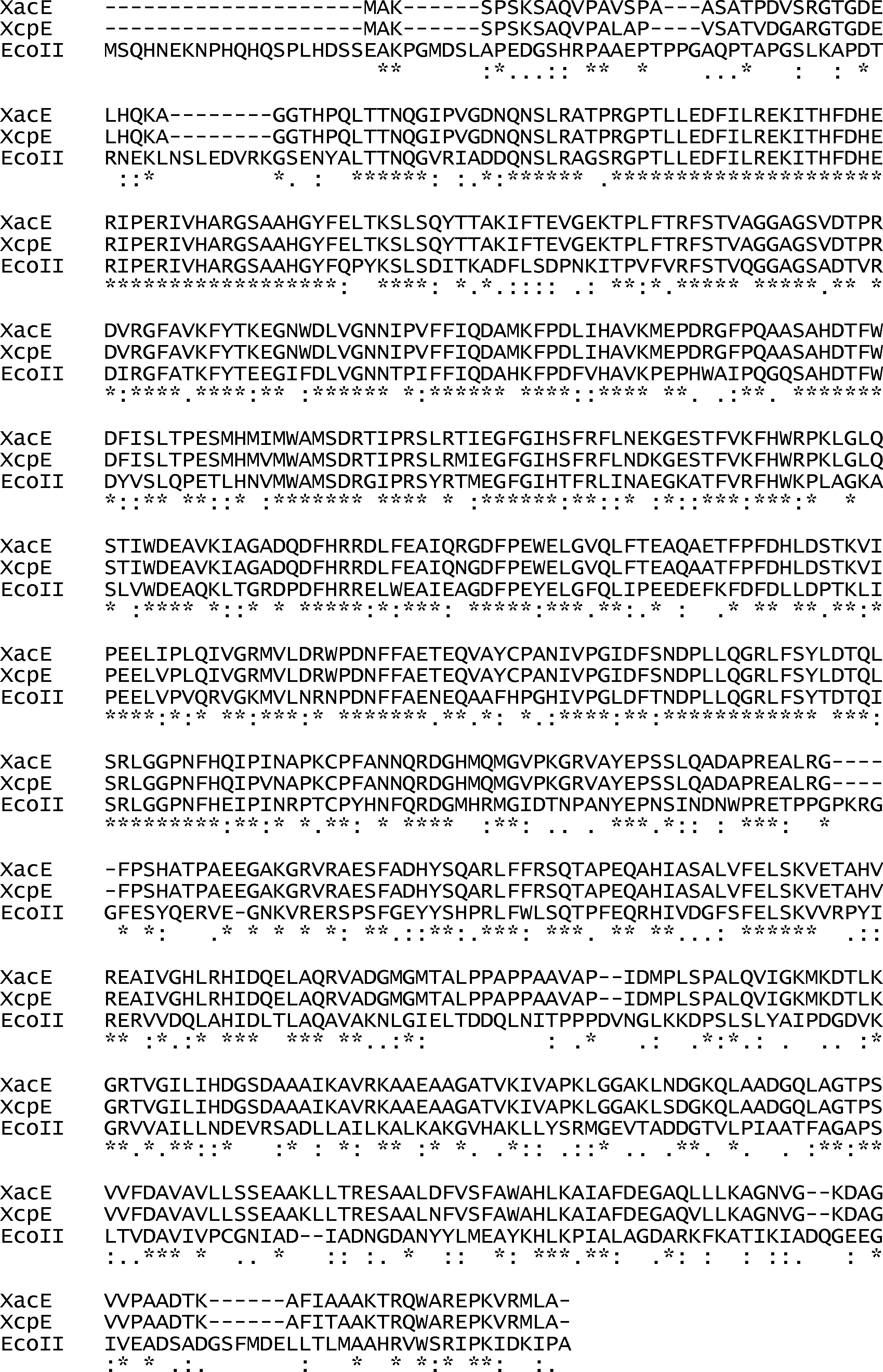

Supplement: Figure S2 — Multiple alignment of the deduced amino acid sequence of Xac KatE (XacE) with catalases KatE of X. campestris pv. phaseoli (XcpE) and HPII of E. coli (EcoII), performed by using ClustalX [26]. An asterisk indicates complete residue conservation, a colon indicates strong group conservation, a period indicates weak group conservation, and a blank space indicates no conservation of residues. (0.47 MB TIF) [file pone.0010803.s003.tif]

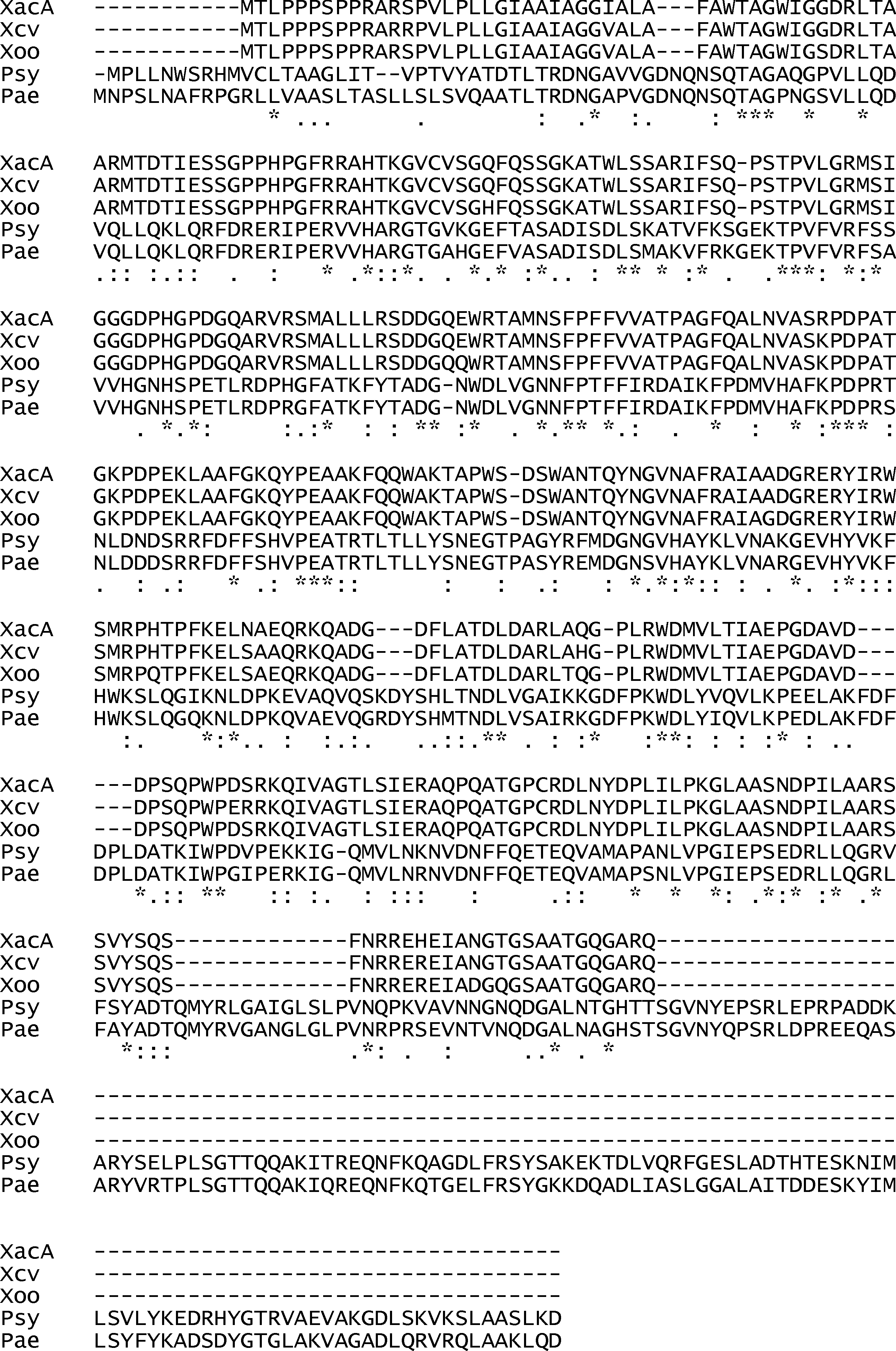

Supplement: Figure S3 — Multiple alignment of the deduced amino acid sequence of Xac SrpA (XacA) with catalases from X. campestris pv. vesicatoria (Xcv), X. oryzae pv. oryzae (Xoo), P. syringae (Psy) and P. aeruginosa (Pae), performed by using ClustalX [26]. An asterisk indicates complete residue conservation, a colon indicates strong group conservation, a period indicates weak group conservation, and a blank space indicates no conservation of residues. (0.45 MB TIF) [file pone.0010803.s004.tif]

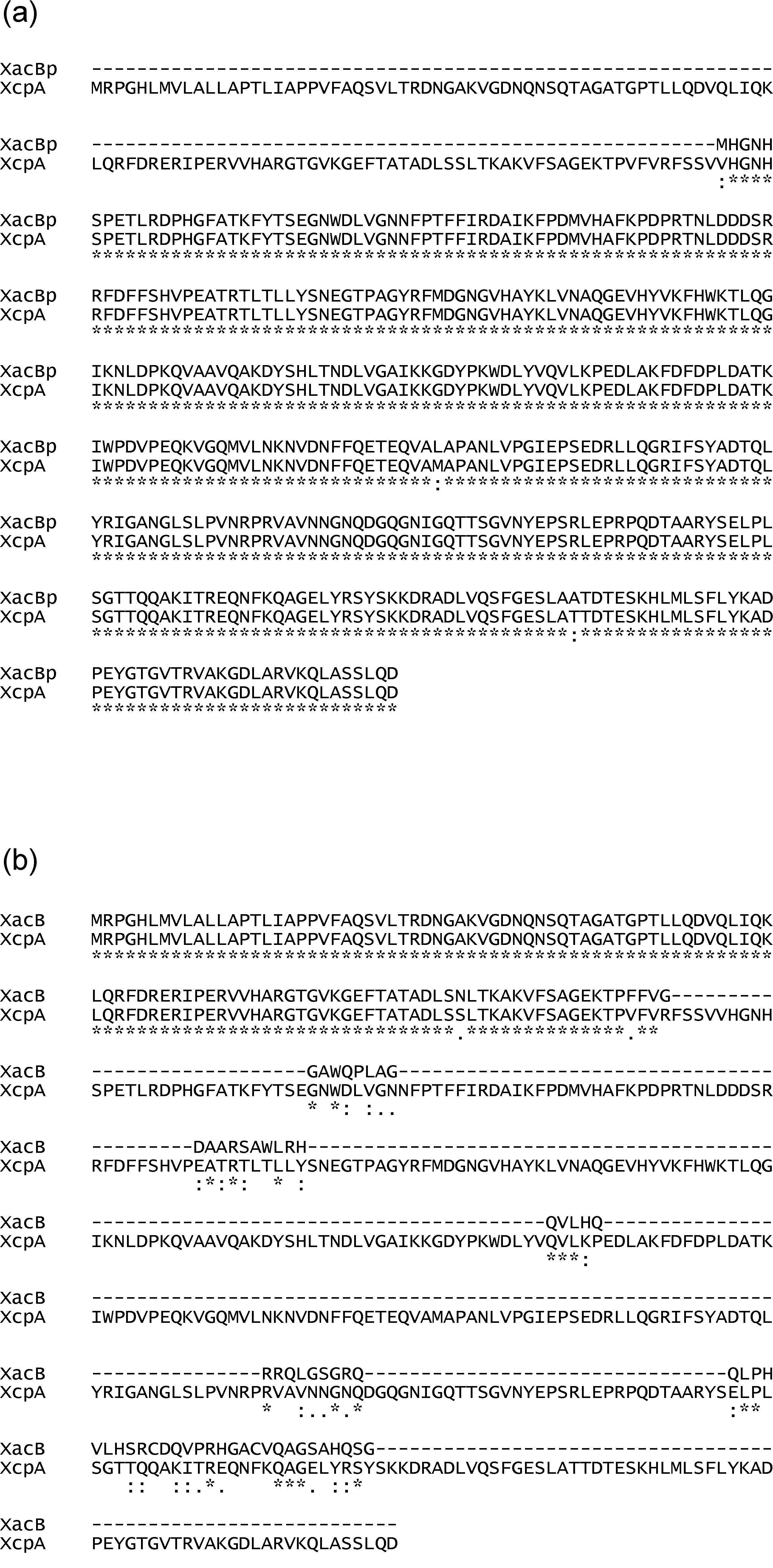

Supplement: Figure S4 — Alignment of the deduced amino acid sequences of Xac CatB precursor (XacBp) (A) and Xac CatB (XacB) (B) with KatA of X. campestris pv. phaseoli (XcpA), performed by using ClustalX [26]. An asterisk indicates complete residue conservation, a colon indicates strong group conservation, a period indicates weak group conservation, and a blank space indicates no conservation of residues. (0.47 MB TIF) [file pone.0010803.s005.tif]

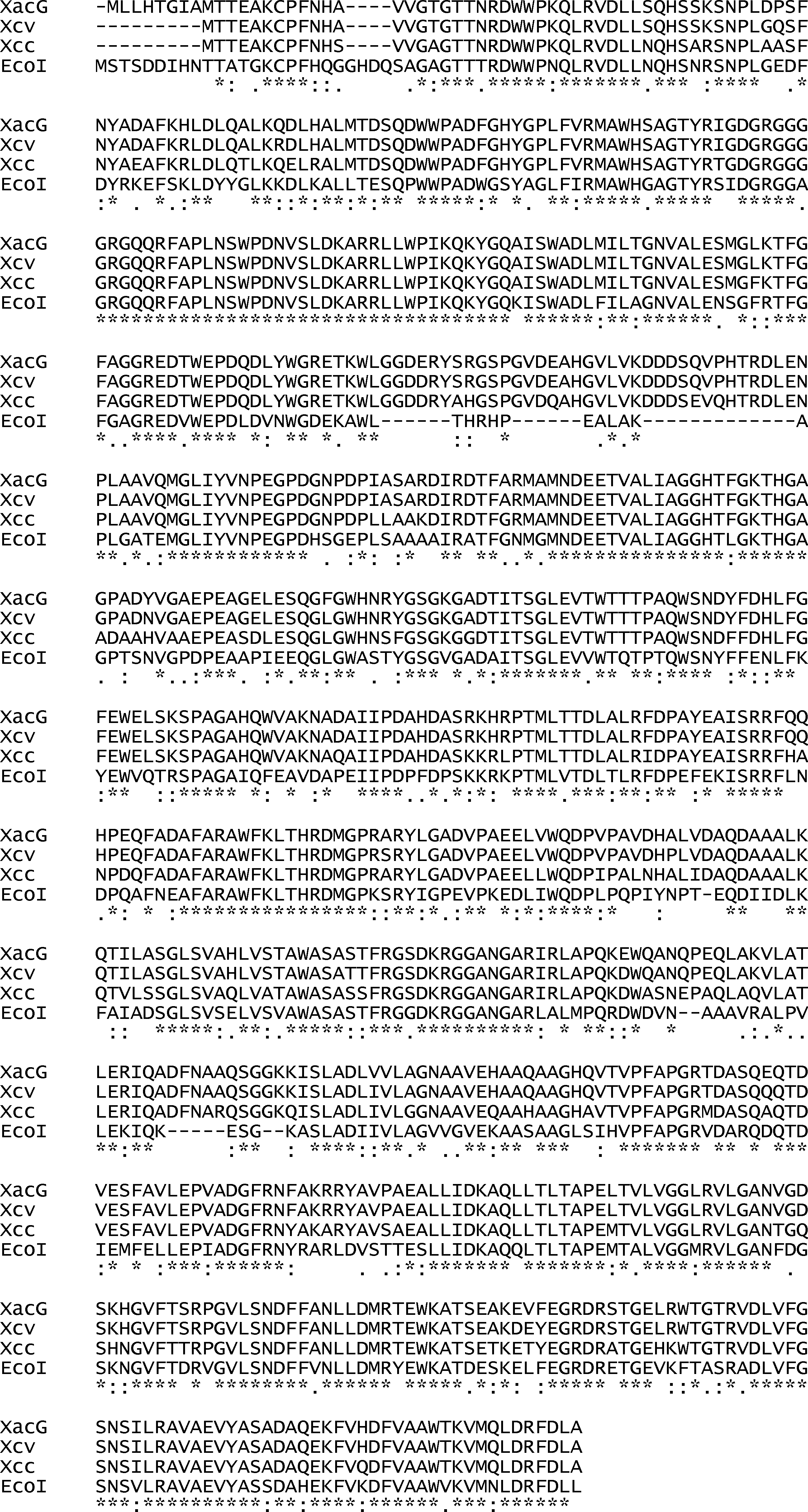

Supplement: Figure S5 — Multiple alignment of the deduced amino acid sequence of Xac KatG (XacG) with catalases from X. campestris pv. vesicatoria (Xcv) and X. campestris pv. campestris (Xcc), and the bifunctional HPI of E. coli (EcoI), performed by using ClustalX [26]. An asterisk indicates complete residue conservation, a colon indicates strong group conservation, a period indicates weak group conservation, and a blank space indicates no conservation of residues. (0.60 MB TIF) [file pone.0010803.s006.tif]

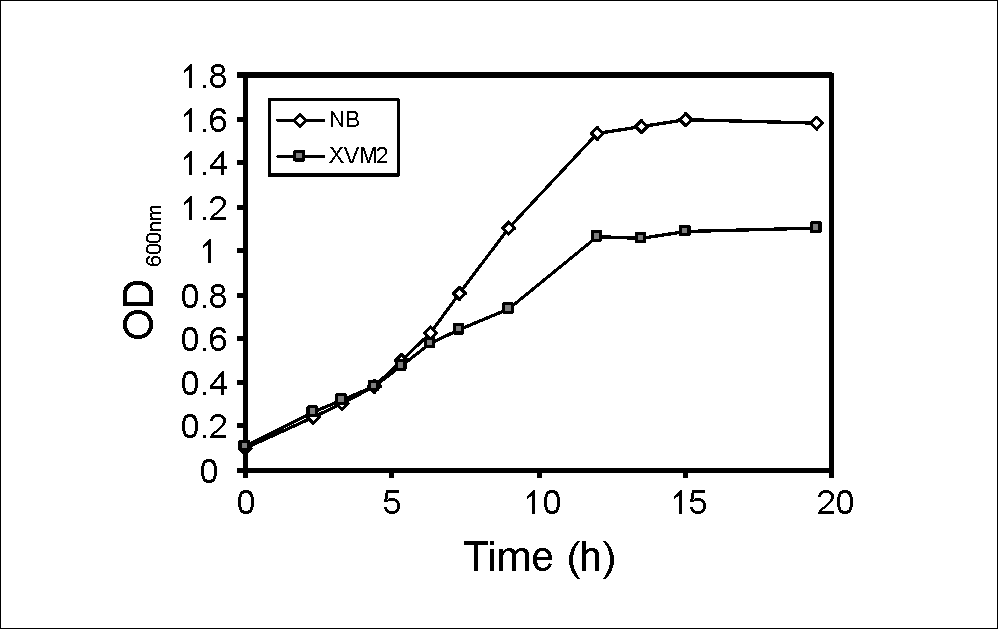

Supplement: Figure S6 — Growth curves of Xac in NB and XVM2 media. Xac cultures were cultivated aerobically in these media at 28°C with shaking at 200 rpm. Aliquots were taken at the indicated times and measured for optical density at 600 nm (OD600). (1.88 MB TIF) [file pone.0010803.s007.tif]
